# Compound developmental eye disorders following inactivation of TGFβ signaling in neural-crest stem cells

**DOI:** 10.1186/jbiol29

**Published:** 2005-12-14

**Authors:** Lars M Ittner, Heiko Wurdak, Kerstin Schwerdtfeger, Thomas Kunz, Fabian Ille, Per Leveen, Tord A Hjalt, Ueli Suter, Stefan Karlsson, Farhad Hafezi, Walter Born, Lukas Sommer

**Affiliations:** 1 Research Laboratory for Calcium Metabolism, Orthopedic University Hospital Balgrist, CH-8008 Zurich, Switzerland; 2 Institute of Cell Biology, Department of Biology, ETH-Hönggerberg, CH-8093 Zurich, Switzerland; 3 Departments for Molecular Medicine and Gene Therapy, Lund University, S-22184 Lund, Sweden; 4 Department of Cell and Molecular, Biology, Section for Cell and Developmental Biology, Lund University, S-22184 Lund, Sweden; 5 IROC, Institute for Refractive and Ophthalmic Surgery, CH-8002 Zurich, Switzerland; 6 Current address: Brain & Mind Research Institute (BMRI), University of Sydney, NSW 2050, Australia

## Abstract

**Background:**

Development of the eye depends partly on the periocular mesenchyme derived from the neural crest (NC), but the fate of NC cells in mammalian eye development and the signals coordinating the formation of ocular structures are poorly understood.

**Results:**

Here we reveal distinct NC contributions to both anterior and posterior mesenchymal eye structures and show that TGFβ signaling in these cells is crucial for normal eye development. In the anterior eye, TGFβ2 released from the lens is required for the expression of transcription factors *Pitx2 *and *Foxc1 *in the NC-derived cornea and in the chamber-angle structures of the eye that control intraocular pressure. TGFβ enhances Foxc1 and induces Pitx2 expression in cell cultures. As in patients carrying mutations in *PITX2 *and *FOXC1*, TGFβ signal inactivation in NC cells leads to ocular defects characteristic of the human disorder Axenfeld-Rieger's anomaly. In the posterior eye, NC cell-specific inactivation of TGFβ signaling results in a condition reminiscent of the human disorder persistent hyperplastic primary vitreous. As a secondary effect, retinal patterning is also disturbed in mutant mice.

**Conclusion:**

In the developing eye the lens acts as a TGFβ signaling center that controls the development of eye structures derived from the NC. Defective TGFβ signal transduction interferes with NC-cell differentiation and survival anterior to the lens and with normal tissue morphogenesis and patterning posterior to the lens. The similarity to developmental eye disorders in humans suggests that defective TGFβ signal modulation in ocular NC derivatives contributes to the pathophysiology of these diseases.

## Background

**Figure 1:**
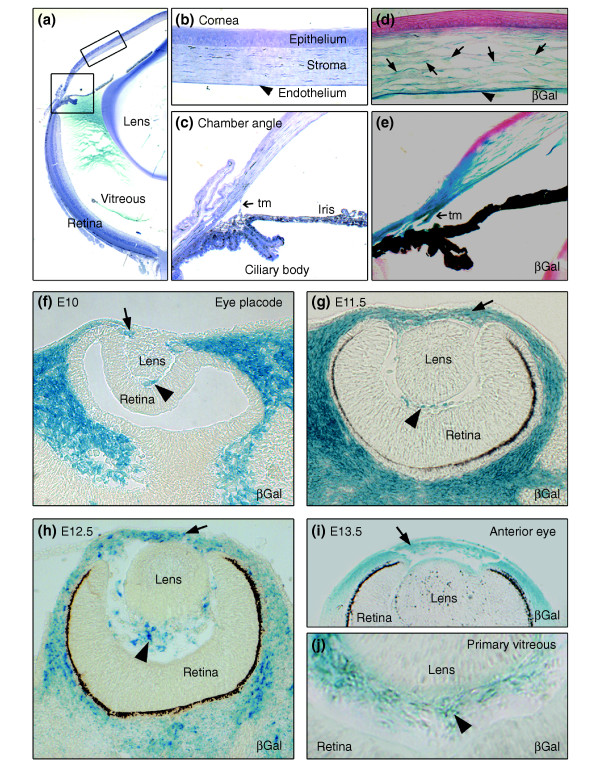
Neural crest (NC)-derived cells contribute to ocular development. **(a) **Toluidine blue staining of an adult eye. The boxed areas correspond to **(b) **a detailed view of the corneal assembly, including outer epithelium, stroma, and inner endothelium, and **(c) **the chamber angle at the irido-corneal transition which includes the trabecular meshwork (tm). **(d-j) ***In vivo *fate mapping of NC-derived, β-galactosidase (βGal)-expressing cells (blue) reveals (d) the NC origin of corneal keratocytes (arrows) and of corneal endothelium (arrowhead). (e) Structures of the chamber angle, including the trabecular meshwork are seen to be NC-derived. (f) At E10, the optic cup is surrounded by NC-derived cells expressing βGal. (g-i) The majority of the cells in the periocular mesenchyme (arrows), which forms the anterior eye segment, are of NC origin, as assessed from E11.5 to E13.5. (j) The primary vitreous at E13.5 (arrowheads) shows a strong NC contribution.

Normal functioning of the eye is dependent on a variety of highly specialized structures in the anterior segment of the eye. These include the cornea and lens, which are necessary for light refraction; the iris, which protects the retina from excess light; and the ciliary body and ocular drainage structures, which provide the aqueous humor required for cornea and lens nutrition and for the regulation of intraocular pressure (Figure 1a–e). Development of these tissues involves coordinated interactions between surface and neural ectoderm and periocular mesenchyme that is derived from the neural crest (NC). Failure of these interactions results in multiple developmental eye disorders, such as Axenfeld-Rieger's anomaly, which consists of small eyes (microphthalmia), hypoplastic irises, polycoria (iris tears), and abnormal patterning of the chamber angle between the cornea and the iris; it is also associated with a high prevalence of glaucoma [1].

Development of the anterior eye segment depends on the proper function of two transcription factors in the periocular mesenchyme, the forkhead/winged-helix factor FOXC1 and the paired-like homeodomain factor PITX2. In humans, hypomorphic and overactivating mutations in either gene leads to Axenfeld-Rieger's anomaly [1], and mutation of either *Foxc1 *or *Pitx2 *in mice results in defective anterior eye-segment formation, similar to that seen in human Axenfeld-Rieger's anomaly [2-4]. Whereas downstream targets of FOXC1 expressed in the eye are supposedly involved in modulating intraocular eye pressure and ocular development [5], PITX2 target genes have been associated with extracellular matrix synthesis and stability [6]. In contrast, the upstream regulators of both FOXC1 and PITX2 remain to be determined. Moreover, the identity of cells expressing FOXC1 and PITX2 during anterior eye patterning is unclear.

It is conceivable that aberrant development of mesenchymal NC cells contributes to the malformations observed in Axenfeld-Rieger's anomaly. Indeed, portions of the anterior eye segment, including corneal endothelial cells, collagen-synthesizing keratocytes, and iris melanocytes, were proposed to originate from the NC [7-9]. The definite contribution of NC, however, has been debated, as most of the data comes from avian models in which ocular development appears to be slightly different from that in mammals [10]. Moreover, mechanisms controlling ocular NC migration and differentiation remain to be elucidated.

Transforming growth factor β (TGFβ) is a candidate factor for the control of ocular NC-cell development. TGFβ signaling is required for the generation of many different non-neural derivatives of the NC [11]. Interestingly, TGFβ signaling during eye development is critical, as ligand inactivation and overexpression lead to defective ocular development in mice [12,13]. In both cases normal development of the anterior eye segment is affected, possibly as a result of impaired NC migration and/or differentiation. In particular, the phenotype upon disruption of the *Tgfb2 *gene recapitulates certain features observed in *Foxc1 *and *Pitx2 *mutant mice. The cellular role of TGFβ signaling in ocular NC development is unknown, however, and a link between TGFβ signaling and activation of the transcription factors FoxC1 and Pitx2 in ocular development has not yet been established [12].

We report here the results of *in vivo *cell-fate mapping to define in detail the contribution of the NC to the forming eye in mice. In addition, we used conditional gene targeting to inactivate TGFβ signaling in NC stem cells and, as a result, in ocular NC derivatives in order to assess the actions of TGFβ on these cells during eye development.

## Results

### Neural-crest cells contribute to multiple structures derived from the eye mesenchyme

NC-cell-specific constitutive expression of β-galactosidase in transgenic mice allows monitoring of NC-cell migration and fate during development *in vivo *[9,14]. This approach was used in the present study to define the ocular structures originating from the NC. *Rosa26 *Cre reporter (*Rosa26R*) mice, which express β-galactosidase following Cre-mediated recombination, were mated with transgenic mice expressing Cre recombinase under the control of the *Wnt1 *promoter. Although *Wnt1 *is not expressed in any structure of the developing eye (see Additional data file 1), it is expressed in the dorsal neural tube, allowing *Wnt1-Cre*-mediated recombination in virtually all NC stem cells [11,15]. In *Wnt1-Cre*/*Rosa26R *double transgenic mice, β-galactosidase-expressing NC-derived cells can be visualized by X-gal staining.

NC-derived cells have previously been proposed to contribute to ocular development in mice after embryonic day (E)12 [10]. Interestingly, we found that NC-derived cells were already detectable at E10 surrounding the optic cup and the lens vesicle (Figure 1f). Until E13.5 (Figure 1f–j), the NC-derived cells were found predominantly in the periocular mesenchyme, whereas the overlying epithelium, the lens, and the retina were consistently X-gal-negative. In addition, we observed that structures of the primary vitreous, located between the lens and the retina, are NC-derived (Figure 1f–j). At later stages (Figure 1d,e), X-gal-positive cells contributed to corneal stroma and endothelium and to structures of the chamber angle at the junction between the cornea and the iris. In mature eyes, the stroma of the iris, the ciliary body, and the trabecular meshwork, as well as cells of the choroid and primary vitreous, are all of NC origin (data not shown). Taken together, these results show that NC-derived cells contribute to eye development as soon as the eye vesicle is formed and, subsequently, to various structures of the maturing eye.

### Multiple ocular anomalies arise from inactivation of TGFβ signaling in NC-derived periocular mesenchyme

**Figure 2:**
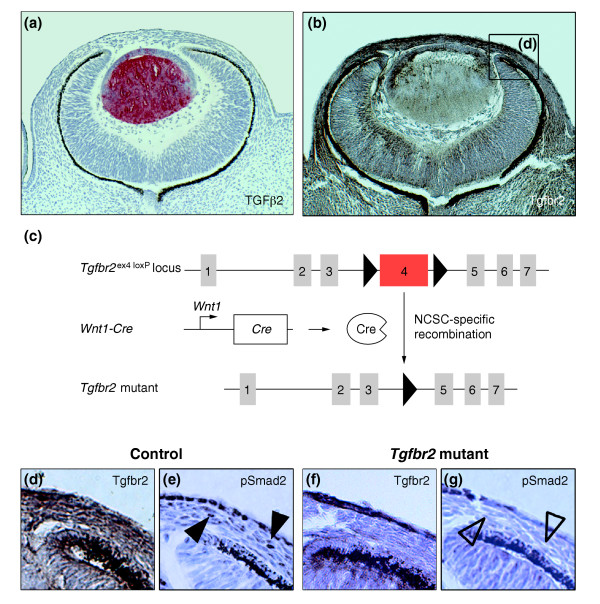
Inactivation of TGFβ signaling in ocular NC-derived cells. **(a,b) **TGFβ ligand and receptor expression in the developing eye at E13.5. (a) Immunoreactive TGFβ2 (red) is predominantly expressed in the lens, whereas (b) Tgfbr2 immunostaining (brown) shows a broad expression of the receptor in the forming eye, including the periocular mesenchyme, lens, primary vitreous, and retina. **(c) **Strategy used for *Cre/loxP*-mediated deletion of exon 4 of the *Tgfbr2 *locus in NC stem cells (NCSC). Exon 4 (red), encoding the transmembrane domain and the intracellular phosphorylation sites of the Tgfbr2 protein, is flanked by loxP sites (triangles) and deleted in NCSCs upon breeding with *Wnt1-Cre *mice. **(d-g) **A detailed view of the forming anterior eye segment (box in b). (d) Strong expression of Tgfbr2 (brown) in the prospective chamber angle, corneal stroma and endothelium can be seen in control embryos. (f) After deletion of *Tgfbr2 *in NCSC, Tgfbr2 is undetectable in corresponding structures. Moreover, defective TGFβ signaling in these structures is also reflected by the absence of phosphorylated pSmad2 in (g) *Tgfbr2 *mutant (open arrowheads) as compared with (e) control embryos (arrowheads).

The expression pattern of TGFβ ligands and their receptors during eye development was visualized by immunohistochemistry at various developmental stages (E10.5 to E18), showing that TGFβ2 expression peaked in the forming lens at E13.5 (Figure 2a) and E15, but decreased towards E18 (data not shown), whereas TGFβ1 and TGFβ3 were undetectable (Additional data file 2 and data not shown). At E13.5, TGFβ receptor type 2 (Tgfbr2) was expressed in periocular mesenchyme, lens, retina, and the primary vitreous (Figure 2b). Because *in vivo *fate mapping revealed a substantial contribution of the NC to the periocular mesenchyme, TGFβ signaling could be important for development of ocular NC derivatives. We therefore analyzed the eyes of mouse embryos after NC-specific inactivation of TGFβ signaling [11,16]. Tissue-specific signal inactivation was achieved by *Wnt1-Cre*-mediated deletion of exon 4 of the *Tgfbr2 *gene (Figure 2c), which leads to loss of Tgfbr2 protein expression in NC stem cells [11]. In such *Tgfbr2*-mutant mice, both Tgfbr2 expression (Figure 2d,f) and TGFβ-induced phosphorylation of the downstream signaling molecule Smad2 (pSmad2; Figure 2e,g) remained undetectable in the periocular mesenchyme.

**Figure 3:**
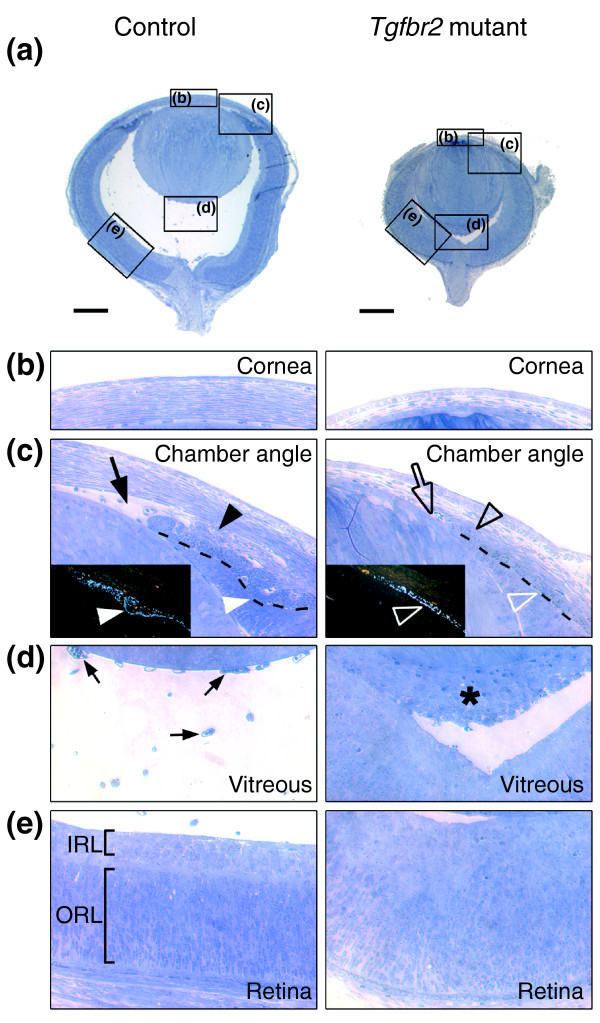
Compound ocular anomalies in *Tgfbr2*-mutant mice. **(a) **Toluidine blue staining of semi-thin sagittal sections of eyes at E18 reveals a smaller size with no anterior chamber and an infiltration of cells behind the lens in *Tgfbr2*-mutant embryos as compared with control embryos. Boxes indicate magnified regions shown in the other panels; scale bars represent 250 μm. **(b) **Abnormal corneal stroma in *Tgfbr2*-mutant embryos. **(c) **Structures of the forming chamber angle, including the trabecular meshwork (black arrowhead) in control eyes are absent in *Tgfbr2*-mutant eyes (open black arrowhead). Here, the lens and the cornea fail to separate to form the anterior eye chamber (open arrow). In addition, dark-field images (insets) visualizing the pigment of the forming iris (broken line in the main image) reveal initiation of ciliary-body formation (white arrowheads) in control eyes and its absence in *Tgfbr2*-mutant eyes (open white arrowheads). **(d) **In control eyes, the primary vitreous consists of loosely arranged vessels of the hyaloid vascular system (arrows). In contrast, *Tgfbr2*-mutant mice show a dense cell mass between the lens and the retina (asterisk), reminiscent of human persistent hyperplastic primary vitreous. **(e) **The retina of control eyes displays typical patterning, with clear separation into an inner layer (IRL) and an outer layer (ORL). In *Tgfbr2*-mutant mice, however, there is no apparent patterning of the retina.

**Figure 4:**
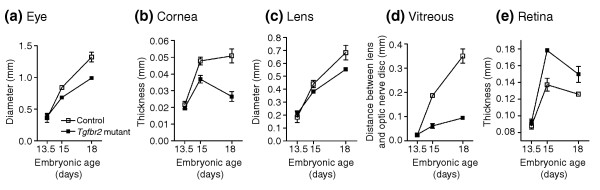
Impaired ocular growth in *Tgfbr2*-mutant mice leads to microphthalmia. **(a) **The developing eyes and **(b-e) **eye compartments of *Tgfbr2*-mutant and control embryos are of comparable size at E13.5, but subsequently, the eyes of *Tgfbr2*-mutant mice are smaller than controls. (c) The growth of the lens is comparable, but (b) the thickness of the cornea and (d) vitreous (measured as the distance between the lens and the optic-nerve disc) are drastically decreased in *Tgfbr2*-mutant mice. (e) In contrast, the thickness of the retina is increased in the mutant. For each time point, mid-organ sagittal sections of both eyes were analyzed for at least three mice.

At E18, main structures of the anterior eye segment, including the forming ciliary body, the iris and the trabecular meshwork, were all well defined in control animals; eye development in the absence of TGFβ signaling in NC-derived cells was therefore analyzed first at E18. Most impressively, eyes from *Tgfbr2*-mutant embryos were 26 ± 1% smaller than eyes from control littermates (Figures 3a, 4). The cornea in control eyes was properly structured into epithelium and endothelium covering a thick stroma, but in *Tgfbr2*-mutant mice the cornea lacked an endothelial layer and no normal stroma was formed (Figure 3b). In control mice, corneal structures and the lens were clearly separated to form the anterior eye chamber; in contrast, cornea and lens of *Tgfbr2*-mutant eyes failed to separate, and no proper anterior eye segment was built (Figure 3c). Moreover, normal formation of the trabecular meshwork and the ciliary body, indicated by a wrinkle in the iris primordium in control eyes, was not observed in *Tgfbr2*-mutant eyes (Figure 3c). In addition, eye sections from E18 *Tgfbr2*-mutant embryos revealed a remarkable accumulation of cells between lens and retina, whereas vessels of the hyaloid vascular system were present in corresponding structures of control eyes (Figure 3d). Finally, the retina of control mice was clearly structured into an inner and an outer layer of cells, whereas the retina of *Tgfbr2*-mutant mice showed diffuse patterning (Figure 3e). Thus, *Tgfbr2*-mutant embryos show microphthalmic eyes with anomalies of the anterior segment, similar to those seen in human Axenfeld-Rieger's anomaly, and the embryos also had defects of the posterior eye segment.

### Persistent hyperplastic primary vitreous in *Tgfbr2*-mutant mice

**Figure 5:**
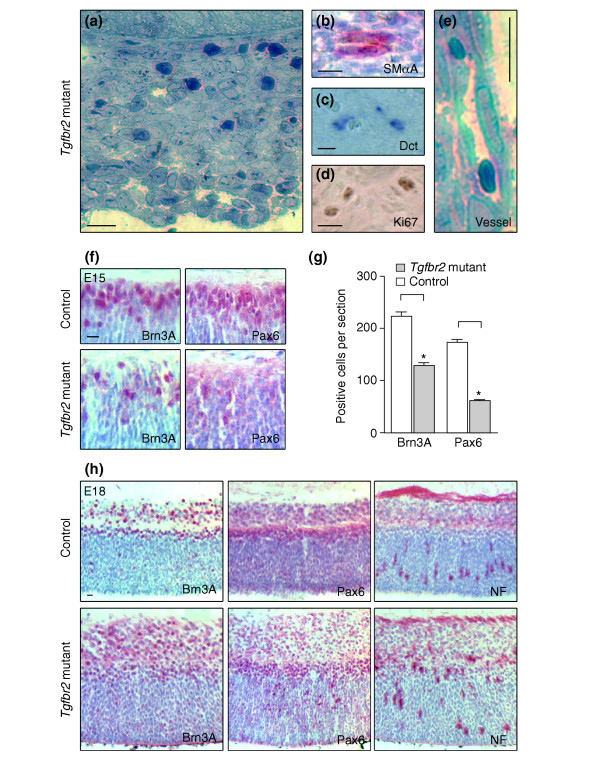
Persistent hypertrophic primary vitreous and disturbed retinal patterning in *Tgfbr2*-mutant mice. **(a) **Detailed view of the persistent hypertrophic primary vitreous in E18 *Tgfbr2*-mutant mice, showing a dense retrolental cell mass. **(b-d) **Staining shows that this mass is composed of various cell types, including (b) smooth muscle α-actin (SMαA)-positive pericytes (red) and (c) prospective melanocytes expressing *Dct *mRNA (blue). (d) Ki67 staining indicates cell proliferation (brown). **(e) **The persistent hypertrophic primary vitreous contains vessels of the hyaloid vascular system. **(f) **Expression of Brn3A and Pax6 (red antibody staining) is readily detectable at E15 in the inner retinal layers of control eyes (top). In *Tgfbr2*-mutant eyes, however, cells expressing these markers are less frequent. **(g) **Bar graph of the results shown in (f). Asterisks indicate a significant difference (*p *< 0.001). **(h) **At E18, staining for Brn3A, Pax6, and neurofilaments (NF) reveals the expected patterning of the retina in control eyes and a diffuse distribution in *Tgfbr2*-mutant embryos. Thus, retinal patterning is disturbed in *Tgfbr2*-mutant embryos with persistent hypertrophic primary vitreous. Scale bars represent 10 μm.

In normal mice, the primary vitreous, including the hyaloid vascular system, persists until postnatal day (P)30. Its regression starts postnatally around P14 to form the avascular and transparent secondary vitreous [17]. In patients with congenital persistent hyperplastic primary vitreous, developmentally abnormal primary vitreous becomes a fibro-vascular membrane, formed behind the lens (retrolentally) [18]. Much as in human persistent hyperplastic primary vitreous [19], irregular retrolental structures present in *Tgfbr2*-mutant mice consisted of several different cell types (Figure 5a–e). These included fibroblast-like cells, prospective melanocytes expressing dopachrome tautomerase mRNA (*Dct*; also called *Trp-2*; Figure 5c), smooth muscle α-actin-positive pericytes (Figure 5b), and vessels of the hyaloid vascular system (Figure 5e). Moreover, staining with an antibody to Ki-67, a protein expressed only in dividing cells, revealed proliferative cells in the retrolental tissue (Figure 5d).

Effects on the retina have been reported in patients with persistent hyperplastic primary vitreous [20]. Moreover, as instructive signals from the lens promote normal patterning of the retina [21], the irregular retrolental structures in *Tgfbr2*-mutant mice might alter normal interaction between the lens and the retina. To test whether retinal development in *Tgfbr2*-mutant mice was affected, retinas from embryos of different ages were immunohistochemically stained for factors known to be expressed at distinct stages of development [22]. At E15, the inner parts of the retina from control mice strongly expressed the transcription factors Brn3A in retinal ganglion cells and Pax6 in amacrine cells of the ganglion cell layers; in contrast, *Tgfbr2*-mutant embryos had lower numbers of both Brn3A- and Pax6-positive retinal cells (Figure 5f,g). Moreover, at E15 the number of cells positive in the TUNEL-staining procedure, which detects apop-totic cells, was higher in the retinas of *Tgfbr2*-mutant embryos than in those of control embryos (13.3 ± 2.5/5 μm section (mutant) versus 5.6 ± 0.5 (control); *p *< 0.01; not shown). At E18, expression of Brn3A, Pax6 and neurofilaments defines distinct layers of the developing retina in control eyes (Figure 5h). In *Tgfbr2*-mutant mice, however, patterning into cell layers was disturbed, and the thickness of the retina was increased in the mutants (Figures 4, 5h). Eyes of *Tgfbr2*-mutant mice are therefore affected by anomalies similar to persistent hyperplastic primary vitreous and by disturbed retinal patterning.

### Expression of Foxc1 and Pitx2, which are both implicated in Axenfeld-Rieger's anomaly, is dependent on TGFβ in NC-derived ocular cells

**Figure 6:**
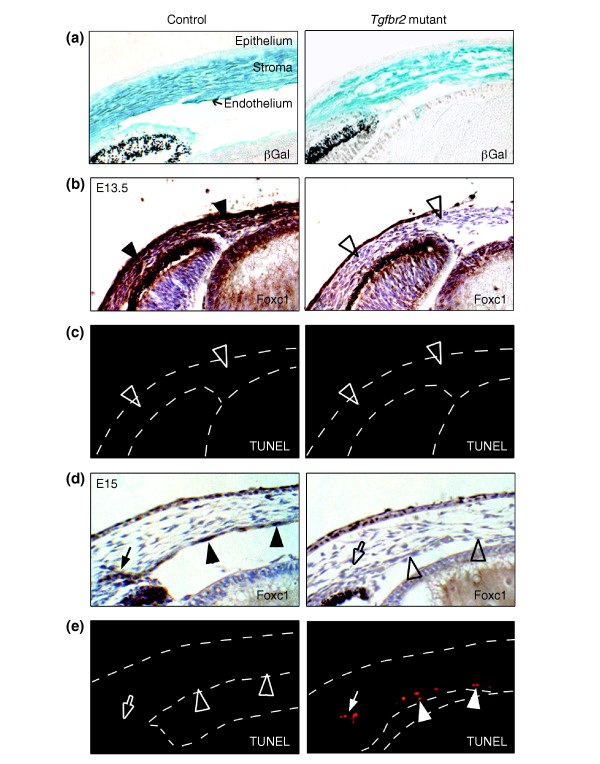
*Tgfbr2*-mutant mice lack corneal expression of the transcription factor Foxc1. **(a) ***In vivo *fate mapping at E15 (βGal, blue) demonstrates that NC-derived cells have correctly migrated into control and *Tgfbr2*-mutant eyes, contributing to corneal stroma and endothelium. **(b) **At E13.5, the periocular mesenchyme of control eyes is positive for Foxc1 antibody staining (brown; arrowheads), whereas Foxc1 is undetectable in corresponding structures of *Tgfbr2*-mutant eyes (open arrowheads). **(c) **No apoptotic cells are found in either control or *Tgfbr2*-mutant eyes at E13.5 by TUNEL analysis (open arrowheads). **(d) **At E15, the eyes of control embryos show strong expression of Foxc1 (brown) in the forming trabecular meshwork (arrow) and in corneal endothelial cells (arrowheads). In *Tgfbr2*-mutant eyes, NC-derived cells localize to the cornea, but Foxc1 is undetectable in prospective endothelial cells (open arrowheads) and in the forming trabecular meshwork (open arrow). **(e) **At E15, cells that fail to express Foxc1 in *Tgfbr2*-mutant eyes appear to undergo apoptosis, unlike in control eyes, as revealed by TUNEL analysis (red).

**Figure 7:**
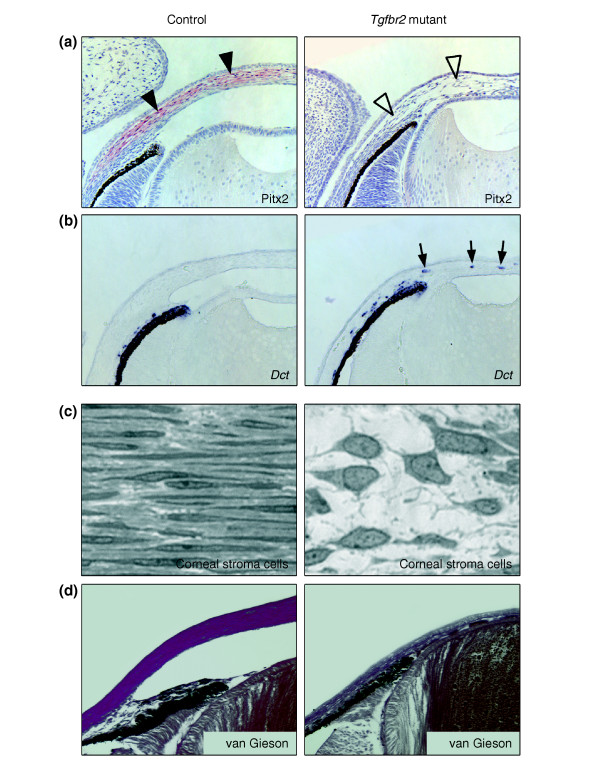
Absence of the transcription factor Pitx2 and of collagen formation in the corneal stroma of *Tgfbr2*-mutant mice. **(a) **At E15, Pitx2 expression, as detected by immunohistochemistry (red; arrowheads) is restricted to corneal stromal cells in control animals but is undetectable in the corresponding structures of *Tgfbr2*-mutant mice (open arrowheads). **(b) ***In situ *hybridization for *Dct*, which marks prospective melanocytes, reveals atypical expression in the corneal stroma of *Tgfbr2*-mutant embryos (arrows). **(c) **High magnification of the corneal stroma shows the typical appearance of thin keratocytes in a parallel orientation and a dense extracellular matrix in control eyes at E18. In contrast, the corneal stroma of *Tgfbr2*-mutant embryos lacks extracellular matrix and has cells with large nuclei and a polygonal shape. **(d) **Van Gieson's staining reveals normal collagen matrix in the corneal stroma of control embryos (purple) that is absent in *Tgfbr2*-mutant embryos.

Anterior eye segment anomalies in *Tgfbr2*-mutant mice were reminiscent of human Axenfeld-Rieger's anomaly (Figure 3). *In vivo *fate mapping revealed that migration of TGFβ-dependent NC cells to the corneal stroma, the endothelium, and the trabecular meshwork was unaffected in *Tgfbr2*-mutant mice (Figure 6a). This indicates that the ocular malformations arise from impaired differentiation rather than from NC-cell migration defects. Interestingly, the anomalies observed in the *Tgfbr2*-mutant embryos recapitulate aspects of ocular defects found in *Foxc1*-null or *Pitx2*-null mice [2,3]. Loss of TGFβ responsiveness in the cells of the periocular mesenchyme might therefore affect expression of the transcription factors Foxc1 and Pitx2. To test this hypothesis, we analyzed eyes from *Tgfbr2*-mutant and control embryos at different developmental stages for the presence of Foxc1 and Pitx2. We confirmed previous reports [2,23] that the two factors are expressed in the periocular mesenchyme during early development (Figure 6b and data not shown); at E15, however, Foxc1 localizes to the corneal endothelium and structures of the forming trabecular meshwork (Figure 6d), and Pitx2 to the corneal stroma (Figure 7a). In contrast, in eyes of *Tgfbr2*-mutant embryos Foxc1 was hardly detectable in the periocular mesenchyme at E13.5 and in the forming chamber angle and corneal endothelium at E15. Moreover, *Tgfbr2*-mutant cells that failed to express Foxc1 appeared to subsequently undergo apoptosis around E15, as revealed by TUNEL staining (Figure 6e).

Pitx2 was strongly expressed in the corneal stroma at E15 in control eyes, but was undetectable in the eyes of *Tgfbr2*-mutant embryos (Figure 7a). Interestingly, some *Tgfbr2*-mutant cells of the corneal stroma expressed *Dct *rather than Pitx2, pointing to incorrect fate acquisition towards melanocytes or misguidance during migration (Figure 7b). At E18, the corneal stroma of control embryos consisted of thin keratocytes organized in a lamellar structure and embedded in extracellular matrix, which provides corneal stability and transparency (Figure 7c). High levels of collagen were detectable in the corneal stroma of control mice, whereas collagen staining was negative in the malformed cornea of E18 *Tgfbr2*-mutant mice, and stromal cells had an abnormal polygonal shape (Figure 7c,d). In summary, NC-derived ocular cells that lack responsiveness to TGFβ fail to express Foxc1 and Pitx2 and fail to undergo correct differentiation into corneal endothelial cells and collagen-synthesizing keratocytes of the corneal stroma.

### TGFβ induces Foxc1 and Pitx2 expression in fibroblasts and in *ex vivo *eye cultures

**Figure 8:**
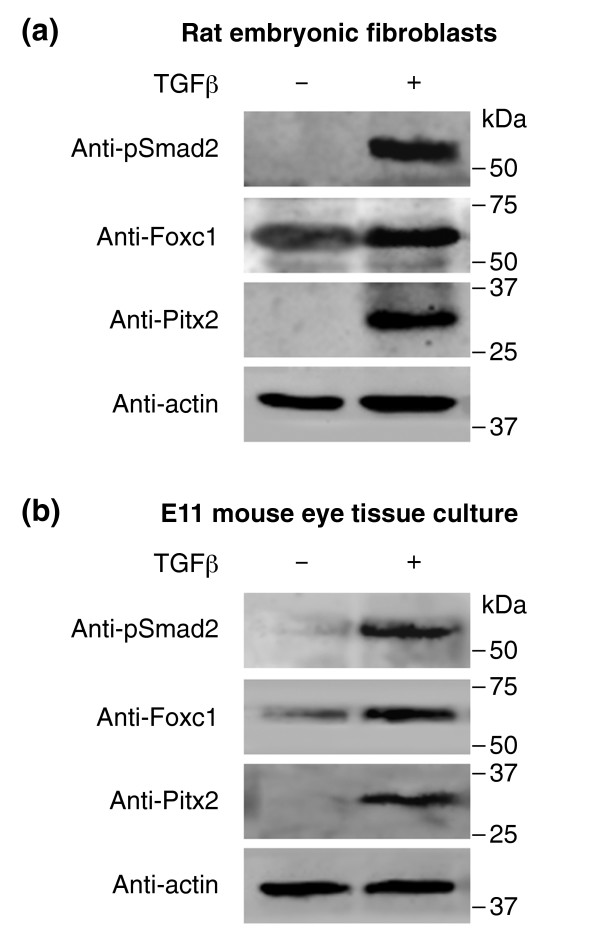
TGFβ regulates expression of Foxc1 and Pitx2. Western-blot analyses of cultured cells were performed using the antibodies shown. **(a) **Rat embryonic fibroblasts were treated with TGFβ, which results in increased levels of phosphorylated pSmad2. Furthermore, TGFβ signaling enhances expression of Foxc1 and induces Pitx2 expression, as revealed by western-blot analysis. **(b) **In *ex vivo *short-term tissue culture of E11 mouse eyes, including periocular mesenchyme, TGFβ strongly upregulates both Foxc1 and Pitx2 expression.

The absence of Foxc1 and Pitx2 expression in the developing eyes of *Tgfbr2*-mutant mice raises the question of whether TGFβ signaling can regulate the expression of Foxc1 and/or Pitx2. To address this issue, cultured rat embryonic fibroblasts were treated with TGFβ and analyzed by western blot for the presence of Foxc1 and Pitx2 (Figure 8a). In the absence of TGFβ, the cells showed weak expression of Foxc1, and Pitx2 expression was undetectable. TGFβ treatment, however, strongly increased Foxc1 expression and induced Pitx2 expression, concomitant with increased levels of pSmad2 (Figure 8a). In addition to fibroblasts, postmigratory NC-derived cells of mouse periocular mesenchyme were also responsive to TGFβ, as shown in short-term tissue cultures of eyes from E11 embryos (Figure 8b): again, treatment with TGFβ resulted in elevated Foxc1 expression. Moreover, Pitx2 expression, which was undetectable in untreated samples, was induced upon addition of TGFβ. In summary, TGFβ treatment upregulates both Foxc1 and Pitx2 expression in a fibroblast cell line and in embryonic eye tissue cultures. TGFβ signaling is therefore not only required for the expression of transcription factors associated with developmental eye disorders, but it is also sufficient to regulate their expression.

## Discussion

**Figure 9:**
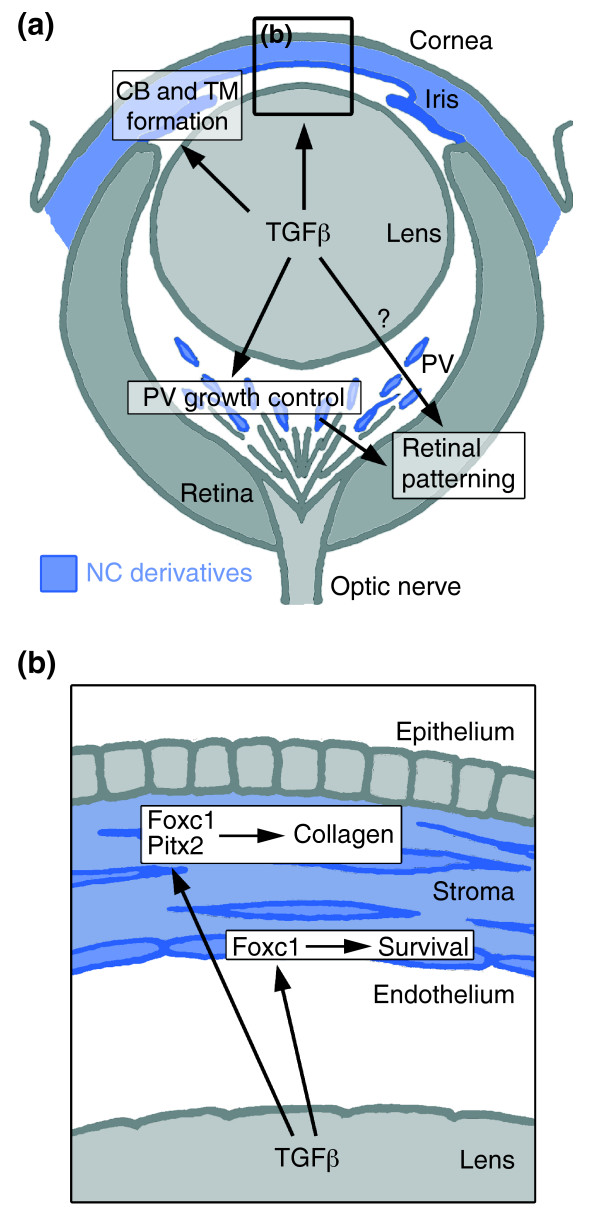
Summary of the TGFβ-dependent development of anterior and posterior ocular structures. **(a) **NC-derived cells (blue) contribute to structures of the anterior eye segment and the primary vitreous (PV). TGFβ signaling is involved in the formation of the ciliary body (CB) and the trabecular meshwork (TM), and in control of PV growth. Moreover, normal PV development and/or TGFβ signaling are important for correct retinal patterning. **(b) **In the cornea, prospective stromal keratocytes and endothelial cells are of NC origin. Here, TGFβ signaling is needed for the expression of the transcription factors Foxc1 and Pitx2 and for normal differentiation of NC-derived cells into collagen-synthesizing stromal keratocytes. Moreover, in forming corneal endothelial cells (and in the TM), expression of Foxc1 and cell survival requires TGFβ signaling.

This study demonstrates that targeted inactivation of TGFβ signaling in NC stem cells perturbs proper development of NC-derived structures in the eye, leading to malformations similar to those found in human Axenfeld-Rieger's anomaly and persistent hyperplastic primary vitreous. The importance of inductive signals from the lens for correct development of the anterior eye segment as well as for retinal patterning has previously been proposed [21,24]. Mutation in genes causing lens anomalies and subsequent abnormal eye formation has further supported this hypothesis [25,26]. Here, we propose that one of the key signaling molecules involved in these processes is TGFβ2, which is highly expressed in the lens at early stages of eye development. Among the signal-receiving cell types, NC-derived cells have a major role in ocular development. According to earlier studies in avian models, NC cells contribute to the developing anterior eye segment [27]. Using *in vivo *fate mapping of NC cells, we have extended these findings to a mammalian model and demonstrate that NC-derived cells contribute to the forming eye as early as the eye vesicle stage. Later, the corneal endothelium, stromal keratocytes and structures of the chamber angle all originate from the NC. In addition, we found a contribution of NC to the primary vitreous, which normally contains a transient network of vessels that supports the inner eye during development. Intriguingly, all these NC-derived tissues fail to develop properly in the absence of TGFβ signaling, although NC-cell migration into the forming eye remains unaffected (Figure 9). Moreover, we show that transcription factors implicated in anterior eye development are targets of TGFβ signaling. Thus, our data indicate that ocular anomalies in mutant mice are due to the absence of a post-migratory response of NC-derived cells to ocular TGFβ.

### NC-cell-specific TGFβ signal inactivation leads to defects of the posterior eye segment

The primary vitreous is situated directly behind the lens and contains the hyaloid vascular system beneath NC-derived cells. Normally, the primary vitreous regresses during postnatal eye maturation through tissue remodeling by apoptosis and phagocytosis, thereby generating the avascular, transparent secondary vitreous [17]. In patients suffering from persistent hyperplastic primary vitreous, a dense cell membrane persists between the lens and the retina. This congenital disorder is often accompanied by cataracts, secondary glaucoma, and a variable degree of microphthalmia [18,28]. Similarly, the primary vitreous in the eyes of *Tgfbr2*-mutant mice appears as a dense cellular membrane, and mutant eyes are smaller than those in control mice. Much as in human persistent hyperplastic primary vitreous [19], the persistent retrolental cell mass in *Tgfbr2*-mutant mice contains fibroblast-like cells, pigmented cells, and vessels of the hyaloid vascular system, and proliferating cells are also seen.

Other mouse mutants have been reported to have a phenotype similar to persistent hyperplastic primary vitreous, including those mutant for the *Arf1, Bmp4*, or *p53 *genes [29-31]. In these models, normal postnatal regression of the primary vitreous fails, resulting in a variable degree of anomalies reminiscent of persistent hyperplastic primary vitreous. Similarly, a dense cell mass in the posterior eye has also been observed previously in *Tgfb2 *null mice, but this was not analyzed further [12]. Treatment of pregnant mice with retinoic acid, which is known to interfere with TGFβ signaling [32], induces anomalies similar to persistent hyperplastic primary vitreous in the offspring [33]. Thus, we conclude that TGFβ signaling in NC-derived cells constituting the primary vitreous is important for tissue morphogenesis.

In the posterior eye segment, retinal development is also disturbed upon ablation of *Tgfbr2 *in NC cells, separately from the generation of persistent hyperplastic primary vitreous. In particular, we observed increased retinal apoptosis at E15 and abrogated retinal patterning, as shown by histology and layer-specific tissue marker expression (Figure 5f–h). Because there is no NC contribution to the retina, this phenotype is probably due to a secondary, non-cell-autonomous effect. The dense persistent primary vitreous in *Tgfbr2*-mutant mice might conceivably constrain instructive signals from the lens to the retina, but such putative signals remain to be identified.

### TGFβ signal-dependent transcription factors and the generation of Axenfeld-Rieger's anomaly

In addition to the defects reminiscent of persistent hyperplastic primary vitreous, all *Tgfbr2*-mutant mice have several developmental defects in the anterior eye. The anterior chamber of the eye is absent in the mutant because the cornea and the lens fail to separate. Furthermore, normal formation of the ciliary body and of the chamber angle with the trabecular meshwork requires TGFβ signaling, as these structures are defective in the mutant mice. The abnormalities presented by *Tgfbr2*-mutant mice are characteristic of the disorders found in patients with Axenfeld-Rieger's anomaly [10]. In this disorder, anterior segment dysgenesis impairs the regulation of the intraocular pressure, which frequently leads to developmental glaucoma.

Other mouse mutants have also been implicated as models for developmental anterior eye disorders. Mice homozygous for an inactivating mutation of *Pax6*, a candidate for human Peter's anomaly, lack eyes [7]. Heterozygous *Pax6*^+/- ^mice have defects in the anterior eye segment, although less severe than those found in *Tgfbr2*-mutant mice [34,35]. The expression of Pax6 in eyes of *Tgfbr2*-mutant mice is not affected, however (data not shown), suggesting that their defects do not depend on Pax6 modulation. In human Axenfeld-Rieger's anomaly, mutations have been found in the genes encoding the transcription factors FOXC1 and PITX2 [1]. Deletion of either *Foxc1 *or *Pitx2 *in mice [2,3] leads to defects in the anterior eye segment, very similar to those in *Tgfbr2*-mutant mice described in this study. In the eye, Foxc1 is expressed in the forming corneal stroma and endothelium and, at later stages, in the structures of the prospective trabecular meshwork [2]. Intriguingly, these structures express Foxc1 in a TGFβ signal-dependent manner, and *Tgfbr2*-mutant prospective corneal endothelial and trabecular meshwork cells undergo apoptosis that is not observed in control eyes. Furthermore, TGFβ upregulates Foxc1 expression in fibroblasts and cultured eye tissue, in agreement with a previous report that described *Foxc1 *as a target gene of TGFβ in human cancer-cell lines [36]. Thus, the data suggest that lens-derived TGFβ signaling controls the survival and development of the NC-derived periocular mesenchyme that gives rise to corneal endothelium and trabecular meshwork by regulating Foxc1 expression in these cells (Figure 9).

Pitx2 is expressed predominantly in NC-derived corneal stromal cells that become collagen-synthesizing keratocytes. In *Tgfbr2*-mutant mice, however, corneal stromal cells do not express Pitx2 and consequently fail to develop into collagen-synthesizing keratocytes. Recently, mutations in the human *TGFBR2 *gene have been reported to cause Marfan's syndrome, a disorder also associated with defective extracellular-matrix synthesis [37]. Thus, we conclude that corneal NC-derived cells must have TGFβ-dependent expression of Pitx2 and differentiation to become stromal keratocytes that produce the collagen matrix (Figure 9). In support of this hypothesis, Pitx2 expression is strongly induced in fibroblasts and eye tissue upon TGFβ signal activation.

In Axenfeld-Rieger's anomaly patients who have a disease-linked mutation in the *PITX2 *gene, ocular anomalies appear to be accompanied by additional defects, including tooth abnormalities, redundant periumbilical skin, and heart defects (all together referred to as Rieger's syndrome) [1]. Apart from its expression in NC-derived cells of the forming eye, Pitx2 is expressed in several other tissues during development, including the teeth, umbilicus, and the heart [23]. In contrast to the mesenchymal expression pattern in the eye, in other organs the expression of Pitx2 is restricted to structures that are not NC-derived, but these structures, and especially the tooth anlagen, are surrounded by or are in close contact with NC-derived cells [14]. Nevertheless, *Tgfbr2*-mutant embryos show no defects in the tooth anlagen or umbilicus at E18 (data not shown). Therefore, Pitx2-dependent anomalies in *Tgfbr2*-mutant mice appear to be restricted to the eyes, although because of embryonic lethality we could not determine whether there are additional Pitx2-dependent defects at a developmental stage later than E19.

We recently reported that inactivation of TGFβ signaling in NC stem cells also leads to cardiac and craniofacial defects and parathyroid and thymic gland anomalies reminiscent of human DiGeorge syndrome [11]. Moreover, depending on the cellular context, TGFβ promotes non-neural cell fates in cultured NC cells [38,39]. Hence, together with the findings from the present study, there is good evidence that TGFβ is a key modulator of non-neural differentiation of post-migratory NC cells during development of multiple tissues, including the eye.

## Conclusion

We have shown an extensive contribution of the NC to the developing anterior eye segment and to the primary vitreous. Moreover, proper differentiation of NC-derived ocular cells is TGFβ-dependent (Figure 9). Specifically, we have shown that TGFβ is involved in growth restriction of the primary vitreous and consequently that *Tgfbr2*-mutant mice suffer from persistent hyperplastic primary vitreous. In the anterior eye segment, anomalies in *Tgfbr2*-mutant mice are reminiscent of human Axenfeld-Rieger's anomaly. Ocular expression of *Pitx2 *and *Foxc1*, which when mutated can cause Axenfeld-Rieger's anomaly, is TGFβ-dependent, suggesting that both transcription factors are involved in mediating TGFβ signaling in ocular cells during development. Interestingly, a report of a family suffering from both Axenfeld-Rieger's anomaly and persistent hyperplastic primary vitreous suggested a common linkage between genes for Axenfeld-Rieger's anomaly and persistent hyperplastic primary vitreous [40]. Thus, our findings may lead to further understanding of the pathophysiology of Axenfeld-Rieger's anomaly and persistent hyperplastic primary vitreous.

## Materials and methods

### Generation of *Tgfbr2*-mutant mice

The generation of mice used in this study has been described before [9,11,16,41]. Briefly, loxP-sites for Cre-mediated recombination were introduced into the mouse *Tgfbr2 *locus flanking exon 4, which encodes the transmembrane domain and is an important part of the functional intracellular domain of the Tgfbr2 protein. Mice expressing the Cre recombinase under the control of the *Wnt1 *promoter and heterozygous for this *Tgfbr2 *'floxed' allele were mated with mice homozygous for the floxed allele. Inactivation of TGFβ signaling in NC-derived cells was achieved in embryos inheriting *Wnt1-Cre *and two *Tgfbr2 *floxed alleles [11]. 100% of all mutant embryos had the phenotype described in this study, as assessed by the analysis of at least three embryos per stage and staining condition. In contrast, littermates lacking the *Wnt1-Cre *transgene or carrying a wild-type *Tgfbr2 *allele expressed Tgfbr2 normally and did not exhibit any overt phenotype, thus serving as control animals. Genotyping was performed as described [11]. All animal experiments were performed on the C57BL/6 background, which has never been associated with genetic mutations causing retinal degeneration.

### Fate mapping of ocular NC-derived cells *in vivo*

The *Rosa26 *reporter (*Rosa26R*) mouse strain expresses β-galactosidase following Cre-mediated recombination [41]. To define the distinct contribution of the NC during ocular development, *Rosa26R *mice were crossed with *Wnt1-Cre *transgenic mice [9]. At least three whole embryos per stage were stained with the β-galactosidase substrate 5-bromo-4-chloro-3-indolyl-β-D-galactopyranoside (X-gal; Sigma, Buchs, Switzerland) and subsequently fixed in 4% paraformaldehyde overnight at 4°C. Subsequently, embryos were embedded in paraffin, sectioned at 7 μm, and dewaxed for mounting with DFX (Fluka, Buchs, Switzerland). Some sections were counterstained with eosin (Fluka).

### Staining procedures

Embedding, sectioning and staining procedures were performed as described [11]. Briefly, the eyes of at least three embryos per stage were stained with primary antibodies to TGFβ1, TGFβ2, and TGFβ3 (Santa Cruz Biotechnology Inc., Santa Cruz, USA), Tgfbr2 (Santa Cruz), pSmad2 (Cell Signaling Technology Inc., Beverly, USA), Ki-67 (Lab Vision (UK) Ltd, Newmarket, UK), Brn3A [42], Pax6 (Chemicon International Inc., Temecula, USA), neurofilament (Chemi-con), Foxc1 (Santa Cruz), Pitx2 [23], and GFAP (Sigma). For visualization the ABC elite Kit (Vector Laboratories Inc., Burlingame, USA) with Metal enhanced DAB (Pierce Biotechnology Inc., Rockford, USA) or AP substrate kit I (Vector) as substrates was used. *In situ *hybridization with digoxigenin-labeled riboprobes to *Dct *was performed as described [9,15]. TUNEL assays were performed following the manufacturer's guidelines (Roche Diagnostics, Basel, Switzerland). Standard protocols were used for tissue processing of semi-thin sections and subsequent toluidine blue staining [43]. The Van Gieson's staining procedure was used to visualize collagen formation in the cornea.

### Assessment of ocular growth

At least three *Tgfbr2*-mutant and control embryos per stage were embedded and sectioned. Mid-organ sagittal sections of both eyes were measured using an Eclipse E600 microscope (Nikon, Tokyo, Japan) equipped with a CCD camera (Kappa, Gleichen, Germany) and the PicEd Cora software version 8.08 (JOMESA, Munich, Germany).

### Culture experiments

Rat embryonic fibroblasts (rat2 cell line; American Type Culture Collection, Mannassas, USA) were cultured in DMEM:F12 medium (Gibco/Invitrogen, Carlsbad, USA) containing 10% fetal bovine serum (Sigma). Following a 60 min incubation in DMEM:F12 medium containing 0.1% bovine serum albumin at 37°C, cells were treated with TGFβ (5 ng/ml) for 90 min at 37°C as described [11]. For short-term tissue-culture experiments, the eyes with periocular tissue were removed from nine embryos at E11 by microdissection. Left and right eyes were pooled separately and kept in DMEM:F12 medium containing 0.1% bovine serum albumin and antibiotics with and without TGFβ (5 ng/ml), respectively, for 6 h at 37°C. Western blot analysis of rat embryonic fibroblast extracts and mouse eye tissue extracts were carried out as described [44]. Primary antibodies used were against Actin (Chemicon), pSmad2 (Cell Signaling), Foxc1 (Santa Cruz) and Pitx2 [23]. Each experiment was performed at least three times.

### Statistics

Results are shown as mean ± standard error of the mean (S.E.M.). Graphs and statistical analyses used Prism 4.01 (GraphPad Software Inc., San Diego, USA).

## Additional data files

The following files are available: Additional data file 1, a figure showing the absence of *Wnt1 *expression during eye formation; and Additional data file 2, a figure showing the expression of TGFβ isoforms during eye formation.

## Supplementary Material

Additional data file 1 A figure showing the absence of *Wnt1 *expression during eye formation Click here for file

Additional data file 2 A figure showing the expression of TGFβ isoforms during eye formation Click here for file
