# Supplementary material for: Compound developmental eye disorders following inactivation of TGFβ signaling in neural-crest stem cells
Source: J Biol. 2005 Dec 14;4(3):11. doi: 10.1186/jbiol29 (PMC1414066; doi:10.1186/jbiol29)
Supplement: Additional data file 1 — A figure showing the absence of Wnt1 expression during eye formation [file jbiol29-S1.pdf]

Additional data file 1

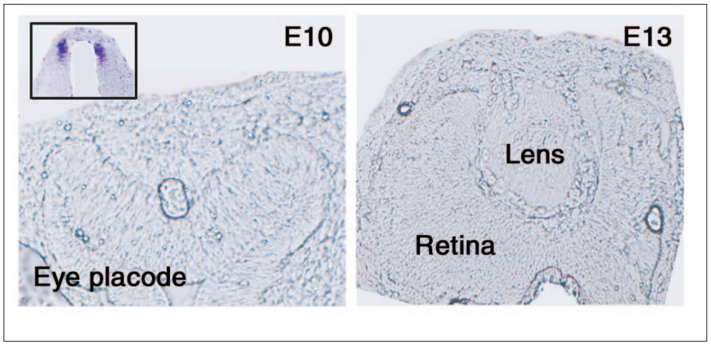

Additional figure 1
Absence of *Wnt1* expression during eye formation. *Wnt1 in situ* hybridization analyses at E10 (left panel) and E13 (right panel) revealed that *Wnt1* expression is readily detectable in the dorsal neural tube (purple; insert), leading to *Wnt1*-Cre-mediated recombination in virtually all neural crest stem cells [11]. On the same sections *Wnt1* is not, however, expressed in ocular structures during development.
