# Supplementary material for: Compound developmental eye disorders following inactivation of TGFβ signaling in neural-crest stem cells
Source: J Biol. 2005 Dec 14;4(3):11. doi: 10.1186/jbiol29 (PMC1414066; doi:10.1186/jbiol29)
Supplement: Additional data file 2 — A figure showing the expression of TGFβ isoforms during eye formation [file jbiol29-S2.pdf]

Additional data file 2

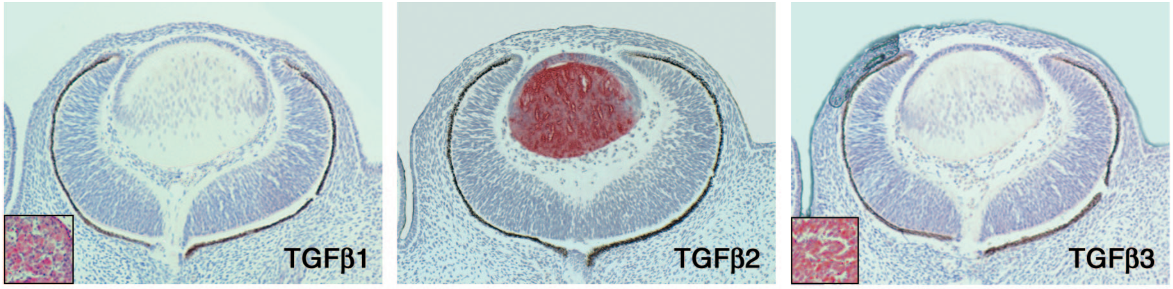

Additional figure 2
Expression of TGFβ isoforms during eye formation. Although TGFβ1 (left panel) and TGFβ3 (right panel) are not expressed in the developing eye, the expression of TGFβ2 (red; middle panel) peaks at E13.5 and is restricted to the lens. Insets serve as controls and illustrate areas in the trigeminal ganglia positive for TGFβ1 and 3 on same sections.
